# Estimation of ischemic core in acute ischemic stroke with CT angiography and non-contrast CT: Attenuation changes in ASPECTS regions vs. automated ASPECTS scoring

**DOI:** 10.3389/fnins.2022.933753

**Published:** 2022-07-26

**Authors:** Jing Li, Yuling Peng, Jiayang Liu, Jiajing Wu, Yunzhuo Yao, Sirun Gu, Zhiwei Zhang, Yi Li, Jingjie Wang, Yongmei Li

**Affiliations:** ^1^Department of Radiology, The First Affiliated Hospital of Chongqing Medical University, Chongqing, China; ^2^Medical Imaging Center, Central Hospital of Shaoyang, Shaoyang, China; ^3^Artificial Intelligence and Clinical Innovation Research, Neusoft Research of Intelligent Healthcare Technology, Co., Ltd., Shanghai, China

**Keywords:** ischemic stroke, reperfusion therapy, Alberta Stroke Program Early CT Score, non-contrast CT, CTA source images, perfusion imaging, ischemic core

## Abstract

**Purpose:**

Reperfusion therapies for acute ischemic stroke due to large-vessel occlusion (AIS-LVO) are highly time-dependent, and large infarction is related to poor outcomes and risk of symptomatic hemorrhage. It is of significance to investigate and optimize the screening means and selection criteria for reperfusion therapies to identify more appropriate patients with better outcomes. This study aimed to compare the performance of attenuation changes vs. automated Alberta Stroke Program Early CT Score (ASPECTS) and using CT angiography (CTA) source images vs. non-contrast CT (NCCT) in distinguishing the infarction extent of ischemic core volumes ≥ 70 ml within different time windows.

**Methods:**

A total of 73 patients with AIS-LVO who received multimodal CT were analyzed. The automated software was used to calculate ASPECTS. Attenuation change was defined as the sum of products of relative Hounsfield unit (rHU) values times weighting factors of all 10 ASPECTS regions. rHU value of each region was the HU of the ischemic side over that of the contralateral. The corresponding weighting factors were the regression coefficients derived from a multivariable linear regression model which was used to correlate regional rHU with ischemic core volumes, because each region in the ASPECTS template is weighted disproportionally in the ASPECTS system. Automated ASPECTS and attenuation changes were both calculated using CTA and NCCT, respectively.

**Results:**

Attenuation changes were correlated with ischemic core volumes within different time windows (Rho ranging from 0.439 to 0.637). In classification of the ischemic core ≥ 70 ml, the performances of attenuation changes were comparable with ASPECTS (area under the curve [AUC] ranging from 0.799 to 0.891), with DeLong’s test (*P* = 0.079, *P* = 0.373); using CTA (AUC = 0.842) was not different from NCCT (AUC = 0.838).

**Conclusion:**

Attenuation changes in ASPECTS regions were correlated with ischemic core volumes. In the classification of infarction volumes, attenuation changes had a high diagnostic ability comparable with automated ASPECTS. Measurement of attenuation changes is not involved in complicated scoring algorithms. This measurement can be used as an available, rapid, reliable, and accurate means to evaluate infarction extent within different time windows. The usefulness of infarction volumes measured by attenuation changes to identify more appropriate patients for reperfusion therapies can be validated in future clinical trials.

## Introduction

Reperfusion therapies, including intravenous thrombolysis (IVT), endovascular treatment (EVT), and IVT plus EVT, are the standard of care and lead to improved clinical outcomes of acute ischemic stroke due to large-vessel occlusion (AIS-LVO). Both IVT and EVT are highly time-dependent and involve selecting patients based on the extent of ischemic damage (Powers et al., 2019). Large infarction extent is in relation to poor functional outcome and the risk of symptomatic hemorrhage. Non-contrast CT (NCCT) and CT angiography (CTA) were used for rapid identification of AIS-LVO, and NCCT was recommended to assess the infarction extent based on one-third of the middle cerebral-artery territory rule or Alberta Stroke Program Early CT Score (ASPECTS) within 4.5 h after symptoms onset for IVT (Hacke et al., 2008) and within 6 h for EVT decisions (Berkhemer et al., 2015; Bracard et al., 2016). The ischemic core volume defined by CT perfusion (CTP) was more accurate in the prediction of final infarct extent and stroke outcome (Parsons et al., 2005; van Seeters et al., 2016) and led to better outcomes with IVT than using NCCT-based means (Bivard et al., 2015). The ischemic core volume of 70 ml was an infarction extent criterion to assist decision-making for IVT (Bivard et al., 2015) and EVT (Albers et al., 2018). However, since treatment benefits declined with time, in guidelines, the CTP imaging was recommended only in late time windows or unknown time of onset or for patients with large infarction extent on NCCT to estimate ischemic core volumes and mismatch between core volumes and clinical deficit (Nogueira et al., 2018) or mismatch between core volumes and penumbra (Albers et al., 2018; Ajčević et al., 2020) for reperfusion treatment selection.

The use of CTP was more time-, cost-, and resource-consuming, and there were many pitfalls and artifacts in contrast bolus injection, perfusion data acquisition, arterial input function selection, and parameters map calculation, which cause that access to and utilization of CTP were not readily available across many stroke centers (Wintermark et al., 2015; Kim et al., 2021). Visual assessment of infarction extent and ASPECTS on CT had limits of interrater and intrarater variability (Grotta et al., 1999; Farzin et al., 2016). It is of clinical significance to investigate and optimize the screening means and selection criteria for reperfusion therapies to identify more patients with better outcomes. Automated calculation of ASPECTS can improve the interrater and intrarater agreement and the early detection of subtle CT changes (Maegerlein et al., 2019). Measurement of attenuation changes, that is Hounsfield unit (HU), is an objective and quantitative means. Moreover, this measurement is not dependent on the ASPECTS scoring algorithm. Therefore, we hypothesized that measurement of attenuation changes in ASPECTS regions can be a more available and reliable approach to determine infarction volumes within different time windows for IVT and EVT decisions.

This study aimed to investigate the correlation of attenuation changes in ASPECTS regions on NCCT and CTA source images with ischemic core defined by CTP within different time windows, to compare the performance of attenuation changes in ASPECTS regions vs. automated ASPECTS scoring, and to compare attenuation changes based on CTA source images vs. NCCT images in distinguishing the infarction extent of ischemic core volume ≥ 70 ml.

## Materials and methods

This cross-sectional study was approved by the local ethics committee (No. 2021-274) and performed in accordance with the ethical standards laid down in the 1964 Declaration of Helsinki and its later amendments. Informed consent was waived after review by the local ethics committee.

### Patients

**FIGURE 1:**
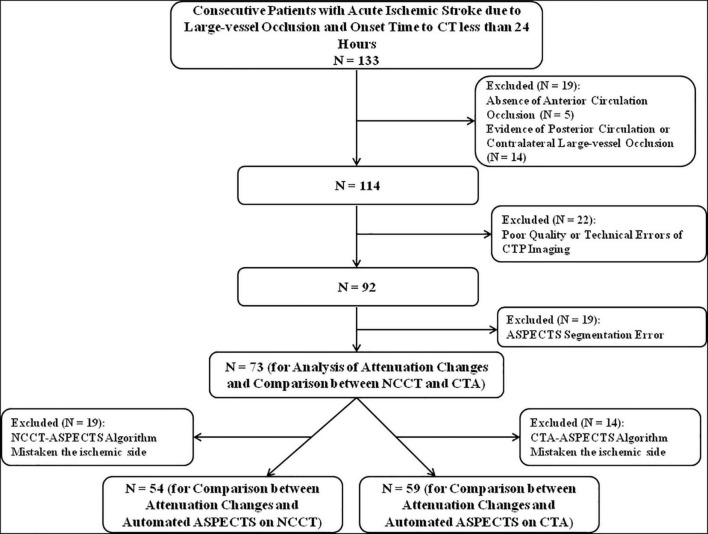
Patient inclusion flowchart.

All patients with AIS due to anterior circulation LVO presented between July 2020 and December 2021 were consecutively screened based on the following inclusion criteria: (1) known stroke onset to CT time and less than 24 h; (2) multimodal CT protocol performed, including NCCT, CTA, and CTP; and (3) with CTA to confirm occlusion of the internal carotid artery or M1 or M2 segment of the middle cerebral artery. The exclusion criteria were as follows: (1) evidence of hemorrhage, tumor, posterior circulation, or contralateral large-vessel occlusion and (2) automated ASPECTS segmentation error, poor quality, or technical errors of CTP imaging. The patient inclusion flow of this study is shown in Figure 1. Clinical characteristics and demographic information were extracted from the medical records.

### Multimodal CT imaging acquisitions

One-stop multimodal CT studies, including NCCT, CTA, and CTP, were performed on a 320-row detector CT scanner (Aquilion ONE, Canon Medical Systems Corporation, Otawara, Japan), using whole-brain dynamic volume intermittent mode. The first scan of this mode started 7 s after the contrast agent injection, followed by continuous intermittent scans at 2-s intervals beginning at 11 s through to 27 s and beginning at 30 s through to 36 s, and then continuous intermittent scans at 5-s intervals beginning at 40 s and end at 50 s or 60 s. This scans mode included 17 or 19 rotations. The scanning speed was 0.75 s/rotation. Initial one scan was performed before any contrast arrival. The dynamic volume scans were performed with the following parameters: 140–160 mm scan coverage, 80 kV, and 310 mA with the initial scan, 300 mA with the expected period of the arterial peak between 17 and 27 s, and 150 mA with the rest of scans. All data were reconstructed with adaptive iterative dose reduction, with a slice thickness of 1.0 mm for CTP studies. The initial scan data were reconstructed with a slice thickness of 5.0 mm for NCCT. One of the scan data of the arterial peak between 17 and 27 s was reconstructed with a slice thickness of 5.0 mm as the CTA source image. The contrast agent (Iopamidol, Bracco Sine, Italy) was injected with a high-pressure syringe by using an individualized technology (P3T Cardiac, MEDRAD, Indianola, PA, United States), which enables the adjustment of contrast volume, injection rate, and injection duration for each individual patient.

### Image analysis

#### CTP evaluation

CTP data were processed using the automated software, Olea Sphere (version 3.0 sp28; Olea Medical Solutions, La Ciotat, France). Arterial input function and venous output function were identified automatically, and perfusion maps were generated based on the deconvolution of the Bayesian probabilistic method (Sakai et al., 2018). Ischemic core volumes were calculated automatically by the default setting with a relative cerebral blood flow less than 25% and time to peak greater than 5 s of that in the contralateral hemisphere. Any area located outside the brain parenchyma and in the subtentorial region was removed from the ischemic core volumes by one radiologist (J.W. with 10 years of experience) who was blinded to the automated ASPECTS and attenuation changes measurements.

#### Automated ASPECTS scoring and attenuation changes in ASPECTS regions

**FIGURE 2:**
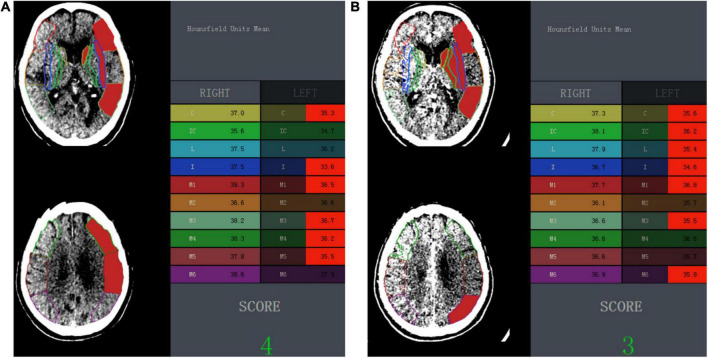
One example illustrating the automated ASPECTS and wHU-ASPECTS value. **(A)** In an 84-year-old man with symptomatic onset to CT time within 4.5 h and CTP-based ischemic core of 176.33 ml, the automated ASPECTS on NCCT image was 4 and wHU-ASPECTS on NCCT was –0.859, both indicated a correct classification of the ischemic extent of ischemic core volume ≥ 70 ml according to the maximum Youden index-associated cutoff value. **(B)** In the same patient, the automated ASPECTS on CTA source image was 3 and wHU-ASPECTS on CTA was –0.746, and both indicated a correct classification of ischemic core size according to the associated cutoff value. ASPECTS indicates Alberta Stroke Program Early CT Score; wHU-ASPECTS, sum of the products of regional relative HU values times corresponding weighting factors.

**FIGURE 3:**
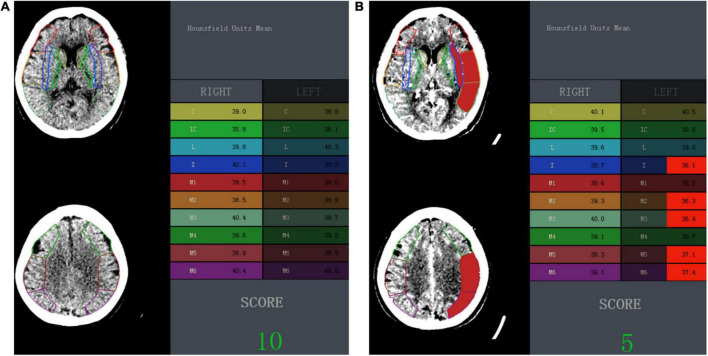
One example illustrating the automated ASPECTS and wHU-ASPECTS value. **(A)** In a 68-year-old woman with symptomatic onset to CT time within 4.5 h and CTP-based ischemic core of 18.31 ml, the automated ASPECTS on NCCT image was 10, and wHU-ASPECTS on NCCT was –0.882, both indicated a correct classification of ischemic core size according to the maximum Youden index-associated cutoff value. **(B)** In the same patient, the automated ASPECTS on CTA source image was 5 and indicated a correct classification, while the wHU-ASPECTS on CTA was –0.764 and failed to make a correct classification. ASPECTS indicates Alberta Stroke Program Early CT Score; wHU-ASPECTS, sum of the products of regional relative HU values times corresponding weighting factors.

Image data of NCCT and CTA were uploaded to a software (NeuBrainCARE, version Beta 1.2.10.34; Neusoft Research of Intelligent Healthcare Technology, Shanghai, China), and automated mapping and segmentation of ASPECTS regions were generated for the calculation of automated ASPECTS and attenuation changes. Automated ASPECTS scores based on NCCT (NCCT-ASPECTS) and CTA source images (CTA-ASPECTS) were calculated, respectively, without human interaction (Figures 2, 3). One radiologist (J.L. with 5 years of experience), who was blinded to the ischemic core calculation, inspected the correct segmentation in all cases.

Attenuation changes in ASPECTS regions were defined as the sum of the products of relative HU (rHU) values times weighting factors across all 10 ASPECTS regions (wHU-ASPECTS) (Figures 2, 3). The wHU-ASPECTS equations are as follows:


(1) $$\text{wHU}-\text{ASPECTS}\,\text{on}\,\text{NCCT}=\sum_{A\,S\,P\,E\,C\,T\,S\,r\,e\,g\,i\,o\,n\,s\,o\,n\,N\,C\,C\,T}\left(\beta_{r\,e\,g\,i\,o\,n}•r\,H\,U_{r\,e\,g\,i\,o\,n}\right)$$

(2) $$\text{wHU}-\mathrm{ASPECTS}\,\mathrm{on}\,\mathrm{CTA}=\sum_{A\,S\,P\,E\,C\,T\,S\,r\,e\,g\,i\,o\,n\,s\,o\,n\,C\,T\,A}\left(\beta_{r\,e\,g\,i\,o\,n}•r\,H\,U_{r\,e\,g\,i\,o\,n}\right)$$

The rHU value of each ASPECTS region was the HU value of the ischemic side over that of the contralateral side. Before the calculation of attenuation changes, the ischemic and occlusion side was known and determined by the standard imaging of CTA, and then was used for the calculation. Voxels with an X-ray attenuation below 21 HUs or above 50 HUs were discarded automatically to eliminate old strokes, cerebrospinal fluid, and calcification. As each region in the ASPECTS template is weighted disproportionally in the ASPECTS scoring system (Phan et al., 2006), a multivariable linear regression model was used to correlate regional rHU values with ischemic core volumes and to generate regression coefficients (Reidler et al., 2019). The regression coefficients of each region (β^region^) were the corresponding weighting factors. The regression coefficients and wHU-ASPECTS calculations are shown in Supplementary Material 1.

### Statistical analysis

Categorical variables were reported as whole numbers and proportions, and numerical and ordinal variables were described using median (interquartile range [IQR]) values. The Spearman correlation test was used to assess the correlation of attenuation changes and automated ASPECTS scores with ischemic core volumes, with Rho value of 0–0.2 as slight, 0.2–0.4 as fair, 0.4–0.6 as moderate, 0.6–0.8 as substantial, and 0.8–1 as almost perfect. Receiver operating characteristic (ROC) analysis was used to assess the performance of attenuation changes and automated ASPECTS in distinguishing the infarction extent of ischemic core ≥ 70 ml. The best discriminative cutoff value and associated sensitivity and specificity were based on the maximum Youden index. An internal validation with 500 bootstrap samples was performed to assess the performance stability of attenuation changes. Two-sided *P* < 0.05 was considered statistically significant. All statistical analyses were performed using SPSS (version 24.0; IBM Corporation, Armonk, NY, United States), MedCalc (version 15.2.2; MedCalc Software, Mariakerke, Belgium), and Empower (R) (X&Y Solutions Inc.)^1^.

## Results

During the study period, 133 patients with LVO-AIS and onset time to CT less than 24 h were identified. A total of 73 patients met all inclusion criteria, and the median age was 72 years (IQR, 65.5–81 years). Of the 73 patients included in this study, 51 (69.9%) patients had the ischemic core volumes smaller than 70 ml, while 22 (30.1%) had the ischemic core volumes equal to or greater than 70 ml. Overall, 39 (53.4%) patients underwent multimodal CT within 4.5 h after the onset of stroke symptoms, while 34 (46.6%) underwent multimodal CT beyond 4.5 h. The demographic and clinical characteristics are shown in Table 1.

**TABLE 1:** Demographic and clinical characteristics of patients.

Variable	All patients (*n* = 73)
Age in years, median (IQR)	72 (65.5–81)
**Sex, n (%)**	
Male	41 (56.2%)
Female	32 (43.8%)
**Stroke onset to CT time, n (%)**	
≤4.5 h	39 (53.4%)
>4.5 h	34 (46.6%)
NIHSS score at admission^a^, median (IQR)	13 (4–18)
**Occlusion location, n (%)**	
ICA	26 (35.6%)
MCA	47 (64.4%)
Ischemic core volume (mL), median (IQR)	30.87 (12.17–77.94)
NCCT-ASPECTS^b^, median (IQR)	8.5 (6.75–10)
CTA-ASPECTS^c^, median (IQR)	7 (5–9)

NIHSS indicates National Institutes of Health Stroke Scale; ASPECTS, Alberta Stroke Program Early CT Score; ICA, internal carotid artery; MCA, middle cerebral artery; NCCT-ASPECTS, ASPECTS based on non-contrast CT images; CTA-ASPECTS, ASPECTS based on CT angiography source images. ^a^Data available from 63 patients. ^b^Data available from 54 patients. ^c^Data available from 59 patients.

### Relationship of attenuation changes in ASPECTS regions on NCCT and CTA source images with ischemic core volumes

The median ischemic core volume was 30.87 ml (IQR, 12.17–77.94). According to the Spearman correlation test, the wHU-ASPECTS on both NCCT and CTA showed a significant correlation with ischemic core volumes (Rho = 0.596, *P* < 0.001; Rho = 0.526, *P* < 0.001) (Table 2). To investigate this relationship in different time windows (within 4.5 h and beyond 4.5 h), we performed further analysis (Supplementary Material 2). All attenuation changes variables showed a significant correlation with ischemic core volumes in different time windows. The wHU-ASPECTS based on NCCT among patients with onset time beyond 4.5 h showed the highest correlation with ischemic core volumes (Rho = 0.637, *P* < 0.001).

**TABLE 2:** Correlation of attenuation changes in ASPECTS regions on NCCT and CTA with ischemic core volumes.

Pair	NCCT/CTA	Rho	*P*
wHU-ASPECTS and Ischemic Core Volume	NCCT	0.596	<0.001
wHU-ASPECTS and Ischemic Core Volume	CTA	0.526	<0.001

ASPECTS indicates Alberta Stroke Program Early CT Score; wHU-ASPECTS, sum of the products of regional relative HU values times corresponding weighting factors.

### Comparison of discriminative power between attenuation changes in ASPECTS regions vs. automated ASPECTS scoring

**FIGURE 4:**
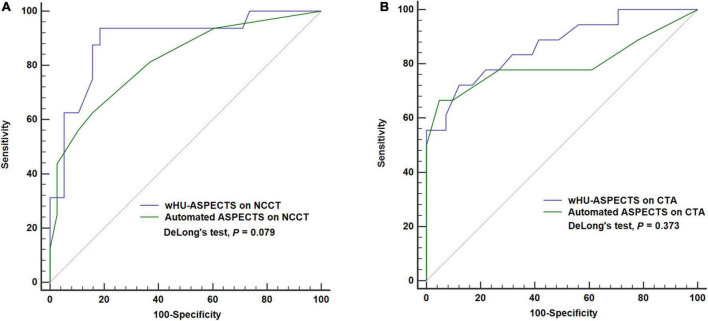
ROC analysis of attenuation changes vs. automated ASPECTS to classify ischemic core volume ≥ 70 ml. **(A)** The wHU-ASPECTS on NCCT image had an AUC of 0.891 (*P* < 0.001), not significantly different from automated NCCT-ASPECTS (AUC = 0.817, *P* < 0.001), with DeLong’s test analysis (*P* = 0.079). **(B)** The wHU-ASPECTS on CTA source image had an AUC of 0.867 (*P* < 0.001), not significantly different from automated CTA-ASPECTS (AUC = 0.799, *P* < 0.001), according to DeLong’s test analysis (*P* = 0.373). ASPECTS indicates Alberta Stroke Program Early CT Score; wHU-ASPECTS, sum of the products of regional relative HU values times corresponding weighting factors.

Among the 73 patients, 19 (26%) patients had a false NCCT-ASPECTS with the ischemic side mistaken. Owing to the same reason, 14 (19.2%) had a false automated CTA-ASPECTS. For the classification of the infarction extent of ischemic core ≥ 70 ml, the wHU-ASPECTS on NCCT showed the area under the curve (AUC) of 0.891 (*P* < 0.001; sensitivity 93.75%, specificity 81.58%), higher than automated NCCT-ASPECTS (AUC = 0.817, *P* < 0.001; sensitivity 62.5%, specificity 84.21%), with a marginal significance according to DeLong’s test analysis (*P* = 0.079); the wHU-ASPECTS based on CTA source images showed the AUC of 0.867 (*P* < 0.001; sensitivity 72.22%, specificity 87.8%), higher than automated CTA-ASPECTS (AUC = 0.799, *P* < 0.001; sensitivity 66.67%, specificity 95.12%), but the difference did not reach statistical significance according to DeLong’s test analysis (*P* = 0.373) (Table 3 and Figure 4). We performed further analysis by different time windows and found similar results (Supplementary Material 3). According to the bootstrap analysis, the AUCs of wHU-ASPECTS on NCCT and CTA were 0.887 (95%CI 0.765–0.964) and 0.856 (95%CI 0.715–0.943), respectively.

**TABLE 3:** ROC analysis of attenuation changes and automated ASPECTS for classification of ischemic core ≥ 70 ml.

	Using NCCT (*n* = 54)		Using CTA (*n* = 59)	
Variable	wHU-ASPECTS	Automated ASPECTS	wHU-ASPECTS	Automated ASPECTS
AUC	0.891 (0.776–0.959)	0.817 (0.688–0.909)	0.867 (0.754–0.942)	0.799 (0.675–0.892)
Youden index	0.753	0.467	0.600	0.618
Cut-off	>−0.874	≤7	>−0.772	≤4
Sensitivity (%)	93.75	62. 5	72.22	66.67
Specificity (%)	81.58	84.21	87.8	95.12

ASPECTS indicates Alberta Stroke Program Early CT Score; wHU-ASPECTS, sum of the products of regional relative HU values times corresponding weighting factors; AUC, area under the curve.

### Comparison of discriminative power between attenuation changes in ASPECTS regions based on CTA source images vs. NCCT images

**FIGURE 5:**
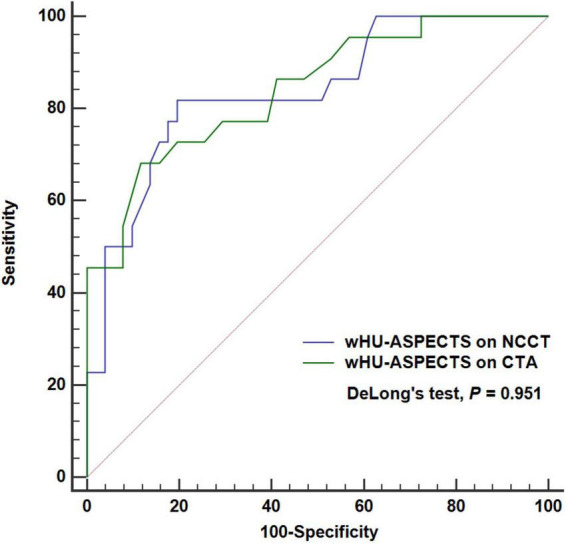
ROC analysis of attenuation changes on NCCT vs. CTA to classify ischemic extent ≥ 70 ml. The wHU-ASPECTS on NCCT image had an AUC of 0.842 (*P* < 0.001), not significantly different from that based on NCCT (AUC = 0.838, *P* < 0.001), with DeLong’s test analysis (*P* = 0.951). ASPECTS indicates Alberta Stroke Program Early CT Score; wHU-ASPECTS, sum of the products of regional relative HU values times corresponding weighting factors.

Among the 73 patients, the wHU-ASPECTS based on CTA source images showed the AUC of 0.842 (95% CI: 0.738–0.917; *P* < 0.001), not significantly different from that based on NCCT (AUC = 0.838, 95% CI: 0.733–0.914; *P* < 0.001) with DeLong’s test analysis (*P* = 0.951), in distinguishing the infarction extent of ischemic core ≥ 70 ml (Table 4 and Figure 5). We performed further analysis by different time windows and found similar results (Supplementary Material 4). According to the bootstrap analysis, the AUCs of wHU-ASPECTS on NCCT and CTA were 0.834 (95%CI 0.718–0.925) and 0.834 (95%CI 0.734–0.919), respectively.

**TABLE 4:** ROC analysis of attenuation changes on NCCT and CTA for classification of ischemic core ≥ 70 ml.

	wHU-ASPECTS on NCCT (*n* = 73)	wHU-ASPECTS on CTA (*n* = 73)
AUC	0.838 (0.733–0.914)	0.842 (0.738–0.917)
Youden index	0.622	0.564
Cut-off	>−0.877	>−0.772
Sensitivity (%)	81.82	68.18
Specificity (%)	80.39	88.24

wHU-ASPECTS indicates sum of the products of regional relative HU values times corresponding weighting factors; AUC, area under the curve.

## Discussion

This study found that attenuation changes in ASPECTS regions on NCCT or CTA had a moderate to substantial correlation with ischemic core volumes within different time windows. The direct comparison analysis showed that the performances of attenuation changes in ASPECTS regions based on CTA (AUC of 0.867) or NCCT (AUC of 0.891) were comparable with automated CTA-ASPECTS (AUC of 0.799) or NCCT-ASPECTS (AUC of 0.817), in distinguishing the infarction extent of ischemic core ≥ 70 ml; the automated ASPECTS scoring was with a rate of about 20% in mistaking the ischemic side, with a lower rate based on CTA source images than that on NCCT images. Therefore, measurement of attenuation change can be used as an accurate, reliable, available, and rapid means to evaluate the infarction volumes of AIS-LVO within different time windows for reperfusion therapies. Moreover, the performance of attenuation changes by using CTA source images was not different from using NCCT. It is feasible and necessary to further investigate the superiority of CTA source images over NCCT in providing information on infarction volumes.

Brain tissue water uptake occurs immediately after arterial occlusion and can be detected by the decrease of CT density or X-ray attenuation (Dzialowski et al., 2004). Water uptake results from intracellular cytotoxic edema, extracellular ionic edema, and extracellular vasogenic edema with subsequent blood–brain barrier breakdown (Simard et al., 2007). Cytotoxic edema and ionic edema precede the stage of vasogenic edema, and the vasogenic edema stage occurs as early as 6 h after stroke (Chen et al., 2021). The degree of water uptake or CT density decrease is associated with the ischemia severity (Kucinski et al., 2004), ischemic edema (water uptake per brain volume) (Broocks et al., 2018a,b), and time of stroke onset (Minnerup et al., 2016). These processes are the underlying pathophysiological mechanisms of our results that attenuation changes in ASPECTS regions were correlated with ischemic core volumes within different time windows. Based on these pathophysiology processes, this study focused on the ischemic core volumes instead of the ischemia severity or the level of CT density decrease, so we included all 10 ASPECTS regions to estimate the ischemic core volumes. In addition, this study used the weighted relative HU as the attenuation changes, because the sizes of regions in the ASPECTS template are not equal to each other and each region has a disproportional weight on the infarction extent in the ASPECTS scoring system (Phan et al., 2006). Measurement of attenuation changes is an objective and quantitative means, not with the issues of interrater and intrarater variability, and this means can improve the capability to detect subtle CT changes in the early time window. Measurement of attenuation changes has the potential to be applied in clinical trials to select patients with AIS-LVO who can benefit from reperfusion therapies, especially in the early time window with only emergency brain imaging recommended.

To the best of our knowledge, this is the first study comparing attenuation changes in ASPECTS regions with automated ASPECTS scoring. In preference to advanced imaging like perfusion studies which are more time- and resource-consuming, the ASPECTS system is used in clinical trials to select eligible patients with AIS-LVO for reperfusion therapies in the early time window (Saver et al., 2015; Bracard et al., 2016). Automated calculation of ASPECTS can improve the interrater and intrarater agreement. Our comparison analysis showed that the measurement of attenuation changes in ASPECTS regions, with substantial discriminative power, was comparable with the automated ASPECTS scoring in distinguishing the ischemic core volumes within different time windows. However, the automated ASPECTS algorithm was with certain false rates of ischemic side detection shown in our study, and this may be due to that the automated algorithm classified each region as either normal or abnormal by HU relevant values and then based on the classified abnormal and normal regions to assess which hemisphere is most likely to be the involved side, while the measurement of attenuation changes is not involved in complicated algorithm and is with the information of ischemic side. Therefore, this measurement is more reliable and readily available for selecting eligible patients for reperfusion therapies in the early time window. This is a critical factor in therapy decision-making in the early time window. ASPECTS system is a 10-point scale; attenuation changes used in our study are continuous data and include all 10 regions, which contains more information regarding ischemic core volumes. We, therefore, hypothesized that measurement of attenuation changes in ASPECTS regions is more accurate than ASPECTS scoring in distinguishing ischemic core size.

Although measurement of attenuation change had higher AUCs than those of automated ASPECTS, these differences did not reach a statistical significance with a trend toward the higher accuracy of attenuation changes. This may be due to the relatively small number of patients included in this study. It is reasonable and feasible to validate these findings in future studies with a larger study sample. Measurement of attenuation changes can identify patients with large infarction volumes who are unlikely to benefit from reperfusion therapies. This measurement can also be used as a surrogate for CTP to estimate the infarction volumes in late time windows and combined with the severity of clinical deficit measured by the National Institutes of Health Stroke Scale to assist decision-making for EVT. NCCT and CTA are the standard imaging means for AIS-LVO and are more time- and resource-saving and readily available than CTP. This measurement can produce an added clinical value to the standard imaging means of NCCT and CTA and can help clinicians make decisions more quickly. This potential has a prominent implication for non-stroke centers where the CTP and EVT management are not available. The faster time period of door-to-recanalization was associated with improved short-term outcomes and a trend toward improved long-term outcomes (Morey et al., 2020). Measurement of attenuation changes can help clinicians rapidly identify patients with large ischemic core or triage patients for transferring to stroke centers capable of CTP and EVT. The usefulness of infarction volumes measured by attenuation changes to identify more appropriate patients for reperfusion therapies can be validated in future clinical trials.

The visibility of ischemic changes and reliability of visual ASPECTS on NCCT are dependent on time from symptom onset (von Kummer et al., 2001; Bal et al., 2015). Some previous studies found that ASPECTS based on CTA source images was more accurate than that on NCCT (Coutts et al., 2004; Puetz et al., 2009; Park et al., 2019), while studies on the effectiveness of attenuation changes on CTA source images are lacking. On CTA source images reflecting reduced blood supply together with water uptake, early ischemic changes are more prominent than those on NCCT images. This may be the cause of our results that the rate of ischemic side mistaken based on CTA source images was less than that on NCCT images. It was speculated that using CTA source images can also improve the performance of attenuation changes in distinguishing the ischemic core volumes, compared to using NCCT. This study found that the discriminative performances by using CTA source images for attenuation changes were not different from using NCCT images within different time windows. It is also worthwhile to further investigate in future studies with a larger study population.

There are several limitations in our study. First of all, 17.8% (13 of 73) of patients had multimodal CT studies after IVT treatment. However, NCCT, CTA, and CTP imaging were performed concurrently for each patient, and this cross-sectional study was designed to correlate CTP data with attenuation changes or ASPECTS on NCCT and CTA. IVT plus EVT is the treatment option for AIS-LVO, so estimation of ischemic core volumes after IVT is also needed. Another limitation was that this study currently focused on patients with AIS-LVO. We aimed to, in future studies, assess the effectiveness of measurement of attenuation changes in AIS not limited to LVO. In addition, ROC analysis and AUC are independent of prevalence. The prevalence of large core should be taken into account in the application of the attenuation changes measure to another population, if one tries to present positive and negative predictive values. The prevalence of large core in our study (22/73, 30.13%) was similar to those in other studies ranging from 18.13% (33/182) (Cao et al., 2021) to 32.65% (16/49) (Bivard et al., 2015; Demeestere et al., 2017; Reidler et al., 2019). Finally, we did not assess the generalizability of attenuation measurement across different scanners. However, the measurement of X-ray attenuation is intrinsically standardized through all CT scanners by calibration to the attenuation coefficient of water, and we used rHU, instead of absolute values, to offer adequate comparability and reproducibility (Minnerup et al., 2016).

## Conclusion

Our study found that attenuation changes in ASPECTS regions on NCCT or CTA had a moderate to substantial correlation with ischemic core volumes defined by CTP in AIS-LVO within different time windows. In the classification of ischemic core volume ≥ 70 ml, measurement of attenuation changes had a high diagnostic ability comparable with the performance of automated ASPECTS. Measurement of attenuation changes is not involved in complicated scoring algorithms. Therefore, this measurement can be used as a readily available, rapid, reliable, and accurate means to evaluate infarction volumes within different time windows. The usefulness of infarction volumes measured by attenuation changes to identify more appropriate patients for reperfusion therapies can be validated in future clinical trials.

## Data availability statement

The raw data supporting the conclusions of this article will be made available by the authors, without undue reservation.

## Ethics statement

The studies involving human participants were reviewed and approved by the Ethics Committee of The First Affiliated Hospital of Chongqing Medical University. Written informed consent for participation was not required for this study in accordance with the national legislation and the institutional requirements.

## Author contributions

JL, JWu, and YML: study concepts and design. JL, ZWZ, and YL: literature research. JL, YLP, JYL, and JWu: data analysis. JL, YZY, and SRG: statistical analysis. JL, YLP, and JWu: manuscript preparation. JL and YML: manuscript editing and review. JWu and YML: guarantor of integrity of the entire study. All authors contributed to the article and approved the submitted version.

## Conflict of interest

YL was employed by Neusoft Research of Intelligent Healthcare Technology, Co., Ltd. The remaining authors declare that the research was conducted in the absence of any commercial or financial relationships that could be construed as a potential conflict of interest.

## Publisher’s note

All claims expressed in this article are solely those of the authors and do not necessarily represent those of their affiliated organizations, or those of the publisher, the editors and the reviewers. Any product that may be evaluated in this article, or claim that may be made by its manufacturer, is not guaranteed or endorsed by the publisher.

## Footnotes

1
www.empowerstats.com
