# Supplementary material for: Estimation of ischemic core in acute ischemic stroke with CT angiography and non-contrast CT: Attenuation changes in ASPECTS regions vs. automated ASPECTS scoring
Source: Front Neurosci. 2022 Jul 26;16:933753. doi: 10.3389/fnins.2022.933753 (PMC9360489; doi:10.3389/fnins.2022.933753)
Supplement: Supplementary file 1 [file Data_Sheet_1.PDF]

Supplementary Material 1

eTable 1. Linear Regression Analysis of ASPECTS Region rHU values and Ischemic Core Volumes

NCCT/CTA, Independent variables	β[a]	*P*	VIF[b]
NCCT			
rHU-C	-0.191	0.100	1.384
rHU-IC	-0.16	0.165	1.367
rHU-L	-0.237	0.108	2.224
rHU-INS	-0.018	0.912	2.855
rHU-M1	0.142	0.285	1.834
rHU-M2	0.045	0.743	1.970
rHU-M3	-0.287	0.014	1.351
rHU-M4	-0.244	0.123	2.559
rHU-M5	0.197	0.264	3.221
rHU-M6	-0.134	0.287	1.626
CTA			
rHU-C	0.042	0.718	1.379
rHU-IC	-0.375	0.002	1.310
rHU-L	-0.283	0.021	1.449
rHU-INS	-0.078	0.606	2.290
rHU-M1	-0.066	0.581	1.451
rHU-M2	0.106	0.495	2.417
rHU-M3	-0.213	0.120	1.866
rHU-M4	0.039	0.767	1.775
rHU-M5	0.182	0.301	3.107
rHU-M6	-0.147	0.290	1.944

rHU indicates relative Hounsfield Units; C, caudate nucleus; IC, internal capsule; L, lentiform nucleus; INS, insula; M1–6, cortical ASPECTS regions

[a]Regression coefficients (β value) of each region are the corresponding weighting factors

[b]The variance inflation factor (VIF) below10 indicates that there are no multicollinearity among variables in regression model.

wHU-ASPECTS Calculation

wHU-ASPECTS on NCCT = (-0.191)*rHU_C + (-0.16)*rHU_IC + (-0.237)*rHU_L + (-0.018)*rHU_INS + (0.142)*rHU_M1 + (0.045)*rHU_M2 +(-0.287)*rHU_M3 + (-0.244)*rHU_M4 + (0.197)*rHU_M5 + (-0.134)*rHU_M6

$$\text{wHU-ASPECTS on CTA} = (0.042)*\text{rHU\_C} + (-0.375)*\text{rHU\_IC} + (-0.283)*\text{rHU\_L} + (-0.078)*\text{rHU\_INS} + (-0.066)*\text{rHU\_M1} + (0.106)*\text{rHU\_M2} + (-0.213)*\text{rHU\_M3} + (0.039)*\text{rHU\_M4} + (0.182)*\text{rHU\_M5} + (-0.147)*\text{rHU\_M6}$$

rHU indicates relative Hounsfield Units; ASPECTS, Alberta Stroke Program Early CT Score; C, caudate nucleus; IC, internal capsule; L, lentiform nucleus; INS, insula; M1–6, cortical ASPECTS regions
