# Supplementary material for: Estimation of ischemic core in acute ischemic stroke with CT angiography and non-contrast CT: Attenuation changes in ASPECTS regions vs. automated ASPECTS scoring
Source: Front Neurosci. 2022 Jul 26;16:933753. doi: 10.3389/fnins.2022.933753 (PMC9360489; doi:10.3389/fnins.2022.933753)
Supplement: Supplementary file 2 [file Data_Sheet_2.PDF]

Supplementary Material 2. Correlation of Attenuation Changes in ASPECTS Regions on NCCT and CTA with Ischemic Core Volumes by Different Time Windows

Time from onset to CT	NCCT/CTA	Rho	*P*
≤ 4. 5 hours (n = 39)	NCCT	0.594	<0.001
	CTA	0.596	<0.001
> 4. 5 hours (n = 34)	NCCT	0.637	<0.001
	CTA	0.439	0.009

ASPECTS indicates Alberta Stroke Program Early CT Score
