# Supplementary material for: Estimation of ischemic core in acute ischemic stroke with CT angiography and non-contrast CT: Attenuation changes in ASPECTS regions vs. automated ASPECTS scoring
Source: Front Neurosci. 2022 Jul 26;16:933753. doi: 10.3389/fnins.2022.933753 (PMC9360489; doi:10.3389/fnins.2022.933753)
Supplement: Supplementary file 3 [file Data_Sheet_3.pdf]

Supplementary Material 3. ROC Analysis of Attenuation Changes and Automated ASPECTS for Classification of Ischemic Core ≥ 70mL by Different Time Windows

Time from onset to CT, NCCT/CTA, and Classification Method	AUC, AUC of bootstrap analysis	Youden Index	Cut-off	Sensitivity (%)	Specifcity (%)
≤ 4. 5 hours					
NCCT (n = 26)					
wHU-ASPECTS	0.899 (0.716–0.982), 0.891 (0.678–0.91)	0.771	>-0.871	88.89	88.24
Automated ASPECTS	0.807 (0.606–0.934)	0.497	≤6	55.56	94.12
CTA (n = 28)					
wHU-ASPECTS	0.922 (0.756–0.989), 0.902 (0.714–0.944)	0.733	>-0.772	90	83.33
Automated ASPECTS	0.811 (0.619–0.933)	0.644	≤4	70	94.44
> 4. 5 hours					
NCCT (n = 28)					
wHU-ASPECTS	0.905 (0.733–0.982), 0.872 (0.695–0.94)	0.81	>-0.874	100	80.95
Automated ASPECTS	0.847 (0.661–0.954)	0.524	≤4	57.14	95.24
CTA (n = 31)					
wHU-ASPECTS	0.804 (0.623–0.924), 0.787 (0.538–0.939)	0.495	>-0.776	62.5	87
Automated ASPECTS	0.783 (0.598–0.91)	0.582	≤4	62.5	95.7

ASPECTS indicates Alberta Stroke Program Early CT Score; wHU-ASPECTS, sum of the products of regional relative HU values times corresponding weighting factors; AUC, area under the curve
