# Supplementary material for: Estimation of ischemic core in acute ischemic stroke with CT angiography and non-contrast CT: Attenuation changes in ASPECTS regions vs. automated ASPECTS scoring
Source: Front Neurosci. 2022 Jul 26;16:933753. doi: 10.3389/fnins.2022.933753 (PMC9360489; doi:10.3389/fnins.2022.933753)
Supplement: Supplementary file 4 [file Data_Sheet_4.pdf]

Supplementary Material 4. ROC Analysis of Attenuation Changes on NCCT and CTA for Classification of Ischemic Core ≥ 70mL by Different Time Windows

Time from Onset to CT, wHU-ASPECTS based on NCCT or CTA	AUC, AUC of bootstrap analysis	Youden Index	Cut-off	Sensitivity (%)	Specifcity (%)
≤ 4. 5 hours (n = 39)					
wHU-ASPECTS on NCCT	0.852 (0.702–0.945), 0.827 (0.648–0.938)	0.615	>-0.877	76.92	84.62
wHU-ASPECTS on CTA	0.873 (0.727–0.958), 0.845 (0.687–0.943)	0.654	>-0.772	76.92	88.46
> 4. 5 hours (n = 34)					
wHU-ASPECTS on NCCT	0.844 (0.679–0.945), 0.833 (0.656–0.946)	0.689	>-0.874	88.89	80
wHU-ASPECTS on CTA	0.802 (0.63–0.918), 0.787 (0.556–0.922)	0.507	>-0.776	66.67	84

wHU-ASPECTS indicates sum of the products of regional relative HU values times corresponding weighting factors; AUC, area under the curve
